# Supplementary material for: Comparison of Bitterness Intensity between Prednisolone and Quinine in a Human Sensory Test Indicated Individual Differences in Bitter-Taste Perception
Source: Pharmaceutics. 2022 Nov 14;14(11):2454. doi: 10.3390/pharmaceutics14112454 (PMC9693378; doi:10.3390/pharmaceutics14112454)
Supplement: Supplementary file 1 [file pharmaceutics-14-02454-s001.zip › pharmaceutics-1987373-supplementary.pdf]

pharmaceutics

Supplementary Materials: Comparison of Bitterness Intensity Between Prednisolone and Quinine in a Human Sensory Test Indicated Individual Differences in Bitter-Taste Perception

Mengyan Deng, Noriko Hida, Taigi Yamazaki, Ryo Morishima, Yuka Kato, Yoshiaki Fujita, Akihiro Nakamura and Tsutomu Harada

Table S1. Primer sequences used for PCR and sequencing.

Gene	Purpose	Primer Sequence	Note
TAS2R19	PCR	CTGGGCTGTAACGAACCATT	Forward
		GCTAGAAGACCCACGATGCT	Reverse
	Sequencing	CTGGGCTGTAACGAACCATT	Detection for SNP rs10772420
TAS2R38	PCR	GGTGGCAACCAGGTCTTTAGATTA	Forward
		ACAGCTCTCCTCAACTTGGCATT	Reverse
	Sequencing	CCAACTAGAGAAGAGAAGTAGAATAGC	Detection for SNPs rs713598, rs1726866, and rs10246939
		ACAAGGCTCAACTGGCAGA	
TAS2R46	PCR	GAGTTGAATCCAGCTTTTAAC	Forward
		ATAGCTGAATGCAATAGCTTC	Reverse
	Sequencing	TTCACTCTGACCCTGATATCTT	Detection for SNP rs2708380

Table S2. Perception of bitter aftertaste of each stimulus (gLMS score) as rated by subjects (n = 50).

	Quinine (0.1 mM)	Quinine (1 mM)	Prednisolone (0.2 mM)	Prednisolone-Saturated Solution	Prednisolone Powder
Mean	2.5	12.9	5.3	9.5	9.6
Min.	0.0	0.7	0.0	0.7	0.7
Max.	12.0	58.7	38.0	44.0	33.3
SD	2.6	13.7	7.4	10.4	8.6
CV	1.0	1.1	1.4	1.1	0.89

Table S3. Relationships between prednisolone-saturated solution bitterness perception and *TAS2R19* genotype. p-values are calculated by Fisher's exact tests.

TAS2R19 Genotype	Strong	Medium or Weak	P-value
GG	23	3	0.0553
AG	12	8	
AA	2	1	
	37	12	

Table S4. Relationships between prednisolone-saturated solution bitterness perception and TAS2R38 phenotype. *p*-values are calculated by Fisher's exact tests.

TAS2R38 Phenotype	Strong	Medium or Weak	*p*-value
PAV/PAV	10	4	1.0000
PAV/AVI	18	6	
AVI/AVI	9	3	
	37	13	

Table S5. Relationships between prednisolone-saturated solution bitterness perception and TAS2R46 phenotype. *p*-values are calculated by Fisher's exact tests.

TAS2R46 Phenotype	Strong	Medium or Weak	*p*-value
W/W	20	5	0.5154
W/*	16	8	
/	1	0	
	37	13	

Table S6. Relationships between 1 mM quinine bitterness perception and TAS2R38 phenotype. *p*-values are calculated by Fisher's exact tests.

TAS2R38 Phenotype	Strong	Medium or Weak	*p*-value
PAV/PAV	8	6	0.0553
PAV/AVI	8	16	
AVI/AVI	9	3	
	25	25	

Table S7. Relationships between 1 mM quinine bitterness perception and TAS2R46 phenotype. *p*-values are calculated by Fisher's exact tests.

TAS2R46 Phenotype	Strong	Medium or Weak	*p*-value
W/W	11	14	0.3961
W/*	14	10	
/	0	1	
	25	25	

Table S8. Bitterness perception (gLMS scores) of the subjects sorted by male and female. *p*-values are calculated by Wilcoxon rank-sum tests.

	Quinine (0.1 mM)			Quinine (1 mM)			Prednisolone (0.2 mM)			Prednisolone-Saturated Solution			Prednisolone Powder		
	Mean	SD	*p*-value	Mean	SD	*p*-value	Mean	SD	*p*-value	Mean	SD	*p*-value	Mean	SD	*p*-value
Male	13.8	11.6	0.1901	38.1	13.7	0.5092	24.6	19.6	0.6978	46.4	19.7	0.6836	32.2	22.4	0.5736
Female	18.0	12.8		42.1	21.6		24.5	13.8		47.2	20.0		36.1	23.9	
